# Characteristics of T-Cell Receptor Repertoire and Correlation With *EGFR* Mutations in All Stages of Lung Cancer

**DOI:** 10.3389/fonc.2021.537735

**Published:** 2021-03-11

**Authors:** Huaxia Yang, Yadong Wang, Ziqi Jia, Yanyu Wang, Xiaoying Yang, Pancheng Wu, Yang Song, Huihui Xu, Dejian Gu, Rongrong Chen, Xuefeng Xia, Zhongxing Bing, Chao Gao, Lei Cao, Shanqing Li, Zhili Cao, Naixin Liang

**Affiliations:** ^1^ Department of Rheumatology and Immunology, Peking Union Medical College Hospital, Chinese Academy of Medical Sciences, Beijing, China; ^2^ Department of Thoracic Surgery, Peking Union Medical College Hospital, Chinese Academy of Medical Sciences, Beijing, China; ^3^ Peking Union Medical College Hospital, Chinese Academy of Medical Sciences, Beijing, China; ^4^ Peking Union Medical College, Chinese Academy of Medical Sciences, Beijing, China; ^5^ Medical Center, Geneplus-Beijing Institute, Beijing, China

**Keywords:** T-cell receptor repertoire, lung cancer, clonality, high-throughput sequencing, epidermal growth factor receptor

## Abstract

Lung cancer is the leading cause of cancer-related deaths worldwide, and its occurrence is related to the accumulation of gene mutations and immune escape of the tumor. Sequencing of the T-cell receptor (TCR) repertoire can reveal the immunosurveillance status of the tumor microenvironment, which is related to tumor escape and immunotherapy. This study aimed to determine the characteristics and clinical significance of the TCR repertoire in lung cancer. To comprehensively profile the TCR repertoire, results from high-throughput sequencing of samples from 93 Chinese patients with lung cancer were analyzed. We found that the TCR clonality of tissues was related to smoking, with higher clonality in patients who had quit smoking for less than 1 year. As expected, TCR clonality was correlated with stages: patients with stage IV disease showed higher clonality than others. The correlation between TCR repertoire and epidermal growth factor receptor (EGFR) status was also investigated. Patients with *EGFR* non-L858R mutations showed higher clonality and a lower Shannon index than other groups, including patients with *EGFR* L858R mutation and wild-type *EGFR*. Furthermore, we analyzed the TCR similarity metrics—that is, the TCR shared between postoperative peripheral blood and tissue of patients with non-distant metastasis of lung cancer. A similar trend was found, in which patients with *EGFR* L858R mutations had lower overlap index (OLI) and Morisita index (MOI) scores. Moreover, the OLI showed a positive correlation with several clinical characteristics, including the tumor mutational burden of tissues and the maximum somatic allele frequency of blood; OLI showed a negative correlation with the ratio of CD4+CD28+ in CD4+ cells and the ratio of CD8+CD28+ in CD8+ cells. In conclusion, TCR clonality and TCR similarity metrics correlated with clinical characteristics of patients with lung cancer. Differences in TCR clonality, Shannon index, and OLI across *EGFR* subtypes provide information to improve understanding about varied responses to immunotherapy in patients with different *EGFR* mutations.

## Introduction

Accumulation of gene mutations and tumor escape from immunosurveillance are the main causes of tumors. Immunotherapy is among the most active research topics in clinical oncology, because it can provide treatments that help prevent the immune escape of tumors. Immune-checkpoint blockers have created enormous interest among cancer immunologists and oncologists (1). Because of the great success of immune-checkpoint blockers in improving survival in patients with lung cancer, more attention has been paid to the mechanism of immune escape, and finding biomarkers that can effectively predict the efficacy of immunotherapy has become an important goal (2–4).

With the wide application of immunotherapies in lung cancer, including as first-line and adjuvant therapies, programmed death ligand 1 and tumor mutational burden (TMB) are no longer sufficient to predict therapeutic outcomes; for adjuvant and neoadjuvant therapy, their predictive efficiency is only 20%–50% in patients with advanced disease (5, 6). T cells, which form a major component of adaptive immunity, are related to immune escape and interact with anticancer treatments (7–9). Therefore, additional investigation of the T-cell receptor (TCR) repertoire could provide more insight into tumor immunity and might provide new biomarkers to predict the efficacy of immunotherapy.

The TCR repertoire, which reflects individuals’ immunity during aging, infections, and even cancer, consists of thousands of TCR clonotypes. The diversity and specificity of TCR are determined by the highly variable complementarity determining region 3 (CDR3) (10). Therefore, distinctively identifiable TCR CDR3 regions can be used to analyze the TCR repertoire. TCR repertoire analysis has potential applications in distinguishing TCR clonality, diversity (Shannon index, richness, etc.), and overlap of unique TCR β-chain sequences identified between tissue and blood [the overlap index (OLI)]. CDR3 clonality and OLI are important tools in cancer diagnosis, therapy, and prognosis (11–13); studies have found that patients with high clonality and high OLI scores respond better to immunotherapy (11, 12) and that patients with significant changes in clonality before and after treatment have poor prognoses (7, 11, 14). Thus, analyzing baseline TCR clonality will enhance our understanding of the mechanisms of anticancer immunity and may provide new predictive biomarkers for anticancer therapies (14). Moreover, the existence of heterogeneity in the TCR repertoire in different types of cancers (15–19) and the intratumor heterogeneity that exists in early-stage lung cancer (20, 21) highlight the need for additional detailed studies of lung cancer in a large population.

Most studies of the TCR repertoire have focused on advanced and localized lung cancer (22, 23). TCR characteristics of early-stage lung cancer in European and American populations have also been reported recently (13). With the use of immunoscores in prognosis assessment of patients with lung cancer and the development of immunotherapy as an adjuvant therapy, it has become necessary to evaluate the TCR repertoire in Chinese patients with early-stage lung cancer (24–26). In this study, we retrospectively conducted a systematic analysis of the CDR3 clonality of the TCR β chain in surgical tissues from Chinese patients with lung cancer to characterize the TCR repertoire of lung cancer.

## Materials and Methods

### Patient Cohorts

Ninety-three patients with lung cancer who received anticancer therapy at the Cancer Center of Peking Union Medical College Hospital (Beijing, China) provided written informed consent for this study. Surgical tissue and postoperative blood were obtained from 74 patients with non-distant metastasis, and needle biopsy results were obtained from 19 patients with advanced lung cancer. Patients with autoimmune disease or AIDS were excluded from the study. Clinical information was collected from the hospital information system and confirmed with the relevant doctors.

### Next-Generation Sequencing–Based Somatic Mutation Detection and Calculation of TMB

Epidermal growth factor receptor (EGFR) status was known for all 93 patients in this study. Tissue and postoperative peripheral blood samples (7–10 days after surgery) from 20 patients with nondistant metastasis were analyzed by next-generation sequencing using a 1,021-gene panel. Genetic analysis was conducted as previously described (27). Briefly, tumor tissues were subjected to genomic tumor DNA extraction using a QIAamp DNA mini kit (Qiagen, Valencia, CA). Circulating tumor DNA was used to prepare sequencing libraries using KAPA DNA library preparation kits (Kapa Biosystems, Wilmington, MA), and genomic DNA sequencing libraries were prepared with Illumina TruSeq DNA library preparation kits (Illumina, San Diego, CA). The libraries were sequenced on a NextSeq CN 500 system (Illumina, San Diego, CA) after hybridization to custom-designed biotinylated oligonucleotide probes (Roche NimbleGen, Madison, WI) targeting 1,021 genes (**Supplementary Table 1**).

Using default parameters, the sequencing data were analyzed and the adaptor sequences and low-quality reads were removed. The clean reads were aligned to the reference human genome (hg19) with the Burrows-Wheeler aligner (version 0.7.12-r1039). GATK (version 3.4-46-gbc02625), MuTect (version 1.1.4), Contra (v2.0.8), and NCsv (in-house) software were used to call variants, copy number variants, and structural variants. Then, variants were filtered by manual verification to exclude synonymous variants, known germline variants in dbSNP, and variants that occurred at a population frequency of greater than 1% in the Exome Sequencing Project.

The TMB was calculated as the number of somatic nonsynonymous single-nucleotide variants and small insertions/deletions per megabase in the coding region (with a variant allele fraction ≥0.03 for tissues) (28, 29).

### High-Throughput DNA Sequencing of TCR β-Chain Genes

TCR sequencing and TCR quantification were performed as in previous research (22, 30, 31). The CDR3 region of the TCR β chain was inclusively and semi-quantitatively amplified by multiplex polymerase chain reaction (PCR), including PCR1 and PCR2. A set of 32 V forward and 13 J reverse primers was used to perform multiplex PCR1 assays to achieve as much amplification as possible of V(D)J combinations. PCR2 universal primers were used in the second round of PCR. The TCR CDR3 region was sequenced using an Illumina HiSeq X ten system, and reads of 151-bp lengths were obtained. Then, the CDR3 sequences were identified and assigned using the MiXCR software package (32).

As previously reported (12), the Shannon index was calculated as follows, where *ni* is the clonal size of the clonotype (i.e., the number of copies of a specific clonotype), *S* is the number of different clonotypes, and *N* is the total number of TCR-cell receptor sequences analyzed:

$$Shannon\;index=-\sum_{i=1}^{S}\frac{ni}{N}ln\frac{ni}{N}$$

T-cell clonality was defined as 1−(Shannon index)/ln(number of productive unique sequences). A maximally diverse population has a clonal score of 0, and a perfectly monoclonal population has a clonality score of 1.

The OLI, the Jaccard index (JI), and the Morisita index (MOI) were used to assess the similarity of TCR repertoires between tumor samples and postoperative blood samples (7–10 days after surgery) from each patient. The metric of TCR repertoire overlap was used to calculate the OLI, as previously reported (11). Briefly, for two samples, a and b, we identified the number of TCR CDR3 sequences present in both samples, along with the sequencing read count of each sequence in each sample. The TCR repertoire overlap between samples was defined as the sum of the sequencing reads from shared TCR sequences divided by the total number of sequencing reads observed in both samples. The JI is also a measure of the similarity in the TCR repertoire between samples, taking into account the specific rearrangements regardless of their respective frequency. MOI is another a measure of TCR repertoire similarity between samples, taking into account the specific rearrangements and their respective frequencies. All metrics range from 0 to 1, in which 1 represents an identical TCR repertoire and 0 represents completely distinct TCR repertoires.

### Flow Cytometry

The protocol for flow cytometry was similar to that used in a previous study (33). Briefly, fresh blood samples were collected from patients 7–10 days before surgical treatment. Ten specific monoclonal antibodies were used to differentiate lymphocyte subsets. First, 100 μL of blood was mixed with the specific monoclonal antibodies and incubated at room temperature for 15 minutes in the dark. The red blood cells in the mix were lysed with the FACS lysing solution (BD Biosciences, San Jose, CA). Then, flow cytometry was used to analyze the residual white blood cells, and the proportions of the lymphocyte subsets were calculated with the FlowJo version 10 data analysis software (FlowJo, Ashland, OR). Lymphocyte subsets were determined by the percentages of total lymphocytes.

### Statistical Analysis

The Mann-Whitney test and a one-way analysis of variance were used to compare differences between groups. Correlations between variables were analyzed using Spearman’s rank test. All statistical analyses were performed using GraphPad Prism 5.0. In this study, all tests were two sided, and p values less than 0.05 were considered statistically significant.

## Results

### Patient Cohorts

The characteristics of the patients are summarized in **Table 1**. As shown, patients were age 27–81 years (median age, 57.81 years; age data not available in two patients), and 38 patients (40.86%) were men. Smoking status was not recorded for 21 patients (22.58%); 59 patients (63.44%) had never smoked. Almost all (12/13) smoking patients had quit. Pathological stages were I, II, or III for 74 patients (79.57%), and disease was stage IV for 19 patients (20.43%). Patients with stages I, II, or III disease were treatment naïve before curative lung resection, and five patients with stage IV disease experienced relapse after several lines of therapy. The main histologic subtype was adenocarcinoma (79.57%).

**Table 1:** Clinical characteristics of 93 patients.

Characteristic	Total No. (N = 93)	%
Median (range) age, years	57.81 (27–81)	97.85
**Sex**		
Male	38	40.86
Female	55	59.14
**Smoking**		
Current/former	13	13.98
Never	59	63.44
NA	21	22.58
**Stage**		
I	60	64.52
II	4	4.30
III	10	10.75
IV	19	20.43
**Histologic subtype**		
Adenocarcinoma	74	79.57
Squamous	9	9.68
Other type	10	10.75

Other type: non–small-cell lung cancer or lung cancer.

NA, not available.

### Correlation Between TCR Repertoire and Clinical Characteristics

**Figure 1:**
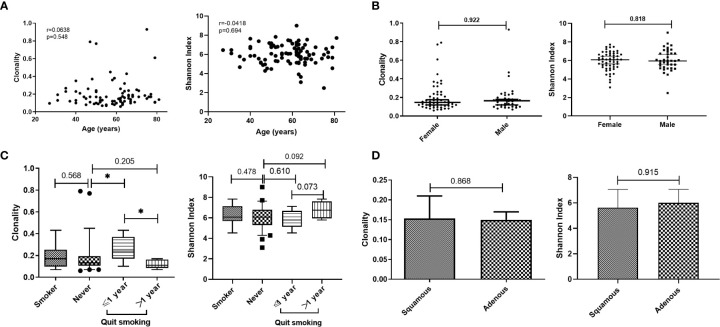
Relationship between T-cell receptor (TCR) repertoire and individual characteristics. **(A)** Correlation between age and clonality or Shannon index. **(B)** Comparison of TCR repertoire between male and female patients. **(C)** Comparison of TCR repertoire between smoking and never-smoking patients. **(D)** Comparison of TCR repertoire between patients with adenocarcinoma (adenous) and squamous carcinoma disease. Statistical analyses were performed using the Mann-Whitney test and Spearman’s rank test. Boxes depict the interquartile range with the line at the median and the whiskers at the 5th–95th percentiles. *p < 0.05.

We retrospectively analyzed the TCR repertoire results from 93 patients with lung cancer. Data from different groups were analyzed to examine whether TCR repertoire was related to clinical characteristics, such as age, sex, smoking history, and histology. The clonality ranged from 0.063 to 0.935 (mean=0.200); the Shannon index ranged from 2.48 to 9.00 (mean=5.98). TCR repertoire, clonality, and Shannon index were not affected by age (**Figure 1A**) and were similar in men and women (**Figure 1B**). No difference was found between current/former smokers and never-smoking patients with respect to TCR repertoire. However, patients who had quit smoking for less than 1 year had higher clonality than those who had quit smoking for more than 1 year or who had never smoked (**Figure 1C**). In our cohort, no correlation was found between TCR repertoire and histology, whether measured by clonality or Shannon index (**Figure 1D**).

**Figure 2:**
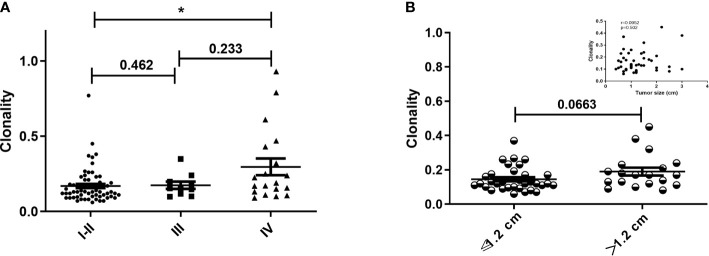
Clonality of T-cell receptor repertoire stratified by pathological stage and tumor size. **(A)** Comparison of clonality among different pathological stages. **(B)** Comparison of clonality in patients with primary tumor diameter greater than or less than 1.2 cm and without lymph node metastasis. Inset shows the correlation between tumor size and clonality in patients without lymph node metastasis. Statistical analyses were performed using the Mann-Whitney test and Spearman’s rank test. *p < 0.05.

Although the characteristics of the TCR repertoire in lung cancer have been extensively studied, there has been limited investigation of the correlation between TCR repertoire and stage. We assessed the relationship between TCR repertoire and pathological stage. Clonality was significantly higher in patients with stage IV versus stage I/II disease; those with stage III disease showed intermediate clonality, which was not significantly different than clonality observed at other stages (**Figure 2A**; **Supplementary Figure 1**). There was no correlation between Shannon index and stage (**Supplementary Figure 3**). Furthermore, among patients with non-distant metastasis, TCR repertoire in patients with versus without lymph node metastasis did not differ (**Supplementary Figures 2** and **3**). We also analyzed the effects of tumor size on TCR repertoire in patients without lymph node or distant metastasis. The stratification by median tumor size (1.2 cm) is shown in **Figure 2B**; clonality was higher when the tumor diameter was greater than 1.2 cm, but not significantly so (p=0.0663); no correlations were found between tumor size and Shannon index (**Supplementary Figure 3**). Because of the high proportion of patients with stage Ia disease in our study, we also analyzed the correlation between Shannon index and tumor size without these patients. There was a negative, and nonsignificant, correlation trend (Spearman r=−0.334; p=0.150; **Supplementary Figure 3**). These results suggest that patients with advanced lung cancer have higher TCR clonality and that the clonality of the TCR repertoire is closely associated with tumor stage.

### Correlation Between TCR Repertoire and *EGFR*

**Figure 3:**
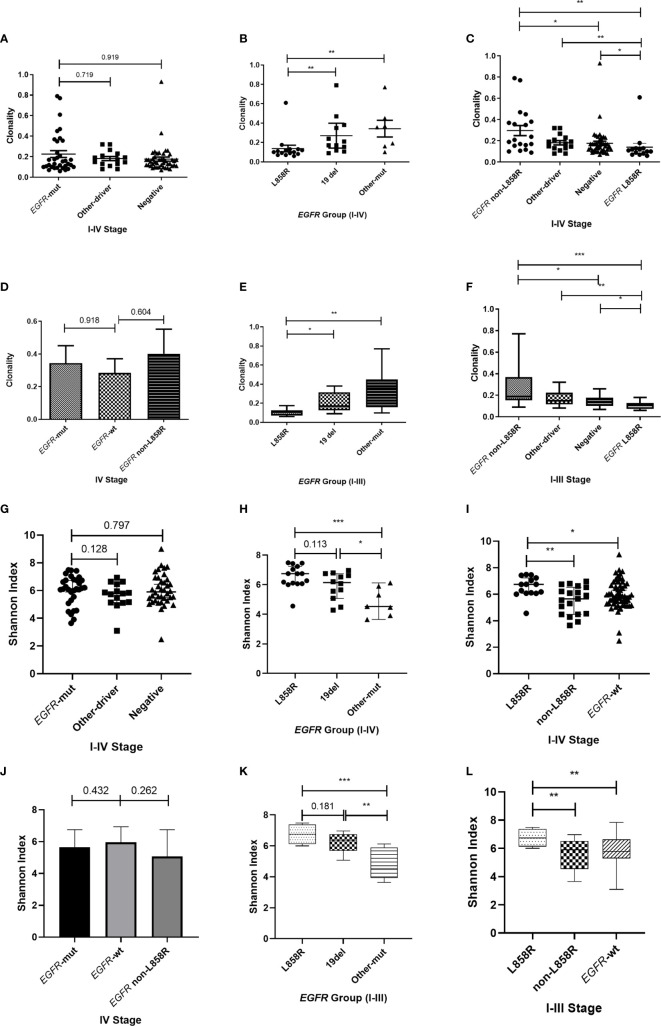
Correlation between T-cell receptor repertoire and *EGFR* mutations. **(A, G)** Comparison of clonality/Shannon index among the three groups. **(B, H)** Differences in clonality/Shannon index within *EGFR* subtypes. **(C, I)** Comparison of clonality/Shannon index between *EGFR* subtype and other groups. **(D, J)** Differences in clonality/Shannon index among three groups of patients with stage IV disease. **(E, K)** Differences in clonality/Shannon index within *EGFR* subtype of patients with stage I–III disease. **(F, L)** Comparison of clonality/Shannon index between *EGFR* subtype and other groups of patients with stage I–III disease. Statistical analyses were performed using the Mann-Whitney test. *p < 0.05, **p < 0.01, ***p < 0.001. wt, wild type.

The impact of EGFR on immunotherapy has been reported in recent studies (34). Studying the relationship between EGFR and the TCR repertoire improves understanding about the application of immunotherapy in lung cancer (13, 35). To assess the relationship between EGFR status and the TCR repertoire, we collected and explored the gene mutations of patients. *EGFR* mutations were detected in 36.5% (34/93) of patients, including 44.1% (15/34) with L858R mutations and 35.3% (12/34) with exon 19 deletions (19del). Patients were divided into three groups according to their mutations (1): *EGFR*-mutated group, consisting of patients with *EGFR*-sensitive mutations; (2) other-driver group, consisting of patients with mutations of genes listed in the National Comprehensive Cancer Network guideline (*KRAS*, *ALK*, *BRAF*, *ROS1*, *ERBB2*, *RET*, *NTRK*) other than *EGFR*; and (3) a negative group, to which the remaining patients were assigned. As shown in **Figure 3A**, the three groups had similar TCR clonalities. The *EGFR*-mutated group was divided into subgroups; patients with *EGFR* 19del and other *EGFR*-sensitive mutations (*EGFR* other mutations) had significantly higher clonality than those with *EGFR* L858R mutations (**Figure 3B**). The subgroup with *EGFR* non-L858R mutations (i.e., those with *EGFR* 19del and *EGFR* other mutations) also had significantly higher clonality than the negative group, and patients with *EGFR* L858R mutations had significantly lower clonality than other groups (**Figure 3C**). All of these differences could be replicated in patients with stage I–III, but not stage IV, disease (**Figures 3C–F**; **Supplementary Figure 4**), possibly because of the limited number of patients with stage IV disease. Similarly, no difference in the Shannon index was found between the *EGFR*-mutated group and other groups (**Figure 3G**). However, the *EGFR* L858R subgroup had a higher Shannon index compared with the *EGFR* non-L858R and *EGFR* wild-type groups (**Figures 3H–L**).

### Similarity of TCR Repertoire Between Postoperative Blood and Tissue in Patients With Non-Distant Metastasis

Because TCR repertoire reflects tumor status, we further analyzed the TCR similarity metric for postoperative peripheral blood and tissue from patients with lung cancer and non-distant metastases. The subset of unique TCR sequences found within each patient’s tumor tissue was evaluated with respect to its detection in the postoperative peripheral blood. By restricting our analysis to the unique TCR sequences found in the tumor tissues, we could explore the correlations between clinical characteristics and TCR repertoire.

**Figure 4:**
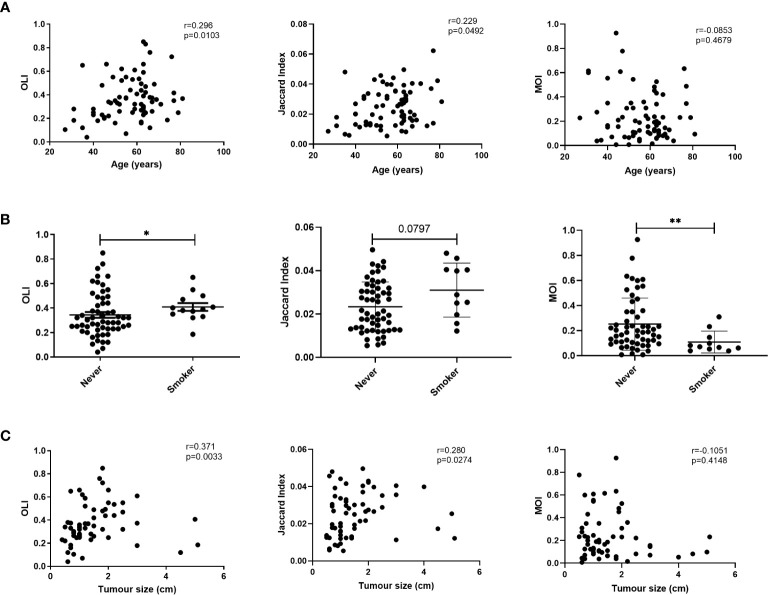
Correlation between T-cell receptor (TCR) similarity metrics and individual characteristics. **(A)** Correlation between age and TCR similarity metrics. **(B)** Comparison of TCR similarity metrics between smoking and never-smoking patients. **(C)** Correlation between TCR similarity metrics and tumor size. Statistical analyses were performed using the Mann-Whitney test and Spearman’s rank test. *p < 0.05, **p < 0.01. MOI, Morisita Index; OLI, Overlap Index.

We evaluated the relationship between age and TCR similarity metrics. Increasing age showed a significant positive correlation with the OLI and JI (**Figure 4A**). Because smoking is a risk factor for lung cancer, we next compared the TCR similarity metrics between smoking and never-smoking patients. Smoking patients showed significantly higher OLI scores and lower MOI scores than never-smokers (**Figure 4B**); a higher JI was noted in smoking patients, but it was not significantly different compared with smokers (p=0.0797). Hence, no correlation between TCR similarity metrics and sex or pathology was identified (**Supplementary Figure 5**).

Given the correlations of clonality with pathological stage, tumor size, and lymph node metastasis, the correlations between these characteristics and TCR similarity metrics were examined. No correlations were found between TCR similarity metrics and stage or lymph node metastasis (**Supplementary Figures S6** and **S7**). We also analyzed the correlation between tumor size and TCR similarity metrics in patients with lung cancer and found a positive correlation between tumor size and the OLI (Spearman r=0.371; p=0.0033) or the JI (Spearman r=0.280; p=0.0274); however, no correlation existed with the MOI (Spearman r=-0.105; p=0.4148; **Figure 4C**). Twenty-two patients had flow cytometry results from preoperative blood. The correlation between flow cytometry and TCR similarity metrics was analyzed. The OLI did not show an association with CD4+ (Spearman r=0.287; p=0.195) or CD8+ (Spearman r=0.287; p=0.195) T cells (**Supplementary Figure 9**). A negative correlation was found between the OLI and the ratio of CD4+CD28+ in CD4+ T cells or the ratio of CD8+CD28+ in CD8+ T cells, as shown in **Supplementary Figure 9**. No correlation between flow cytometry and the JI or MOI was found.

**Figure 5:**
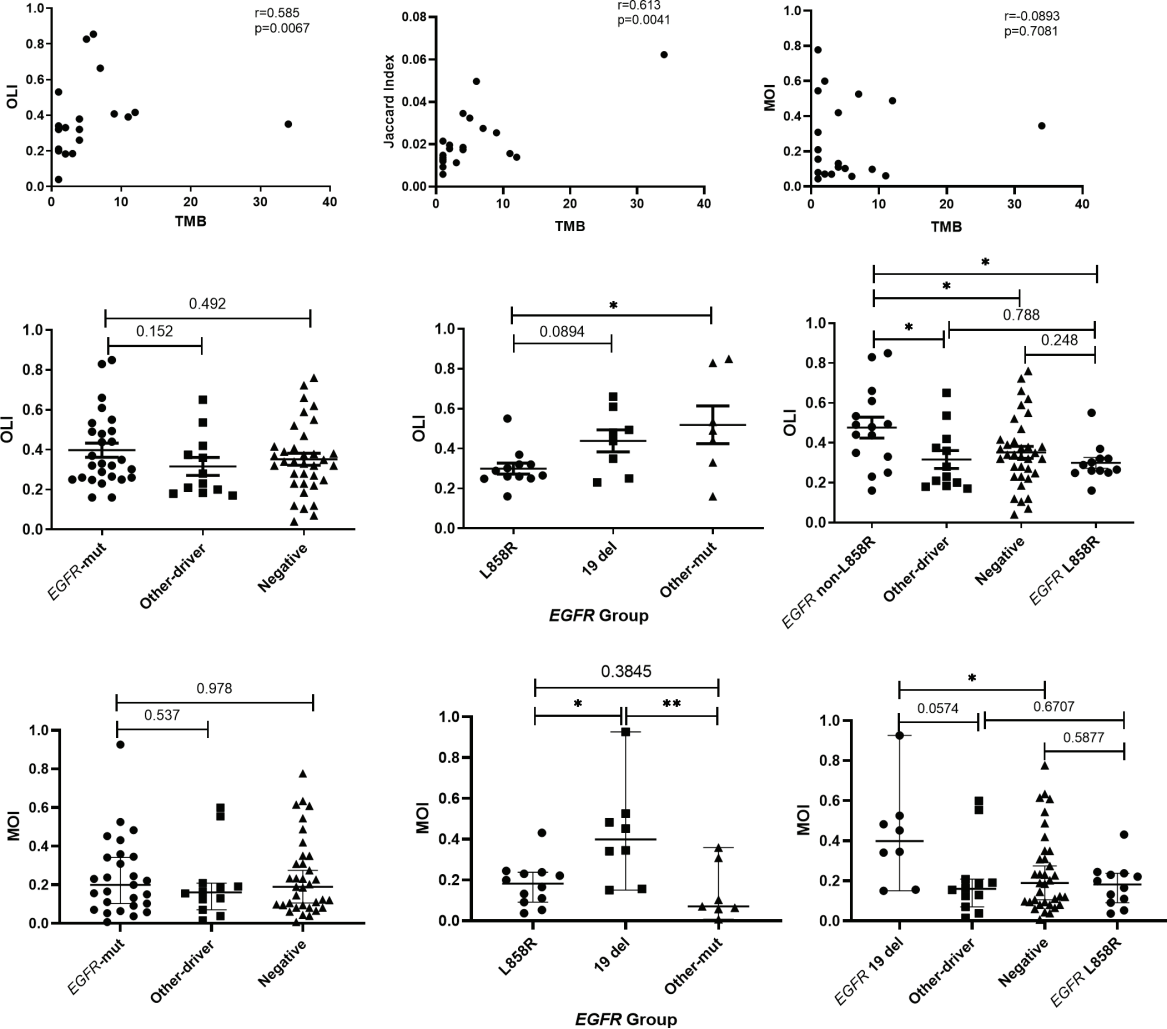
Relationships of T-cell receptor (TCR) similarity metrics with molecular characteristics. **(A)** Correlation between TCR similarity metrics and tumor mutation burden (TMB) of tissues. **(B)** Differences in Overlap Index (OLI) among *EGFR*, other-driver, and negative groups, and differences in OLI among *EGFR* subtypes. **(C)** Differences in Morisita Index (MOI) among *EGFR*, other-driver, and negative groups, and differences in MOI among *EGFR* subtypes. Statistical analyses were performed using the Mann-Whitney test and Spearman’s rank test. *p < 0.05, **p < 0.01. Mut, mutation.

We also analyzed the relationship between TCR similarity metrics and the TMB. The TMB was significantly positively correlated with the OLI (Spearman r=0.585; p=0.0067) and the JI (Spearman r=0.613; p=0.0041; **Figure 5A**). Because the samples were postoperative peripheral blood, mutations were detected in only six of the 18 samples. Although the blood TMB was not significantly correlated with TCR similarity metrics, the OLI was significantly correlated with the maximum somatic allele frequency of gene mutations in the blood, assuming that the undetected maximum somatic allele frequency was 0 (**Supplementary Figure 9**). Furthermore, the correlation between TCR similarity metrics and *EGFR* mutation was analyzed. Like the earlier results, no difference in the OLI or MOI was observed among the *EGFR*-mutated, other-driver, and negative groups (**Figures 5B, C**), but the *EGFR*-mutated group had a higher JI than the other groups (p=0.015 vs. other-driver; p=0.017 vs. negative groups; **Supplementary Figure 10**). The *EGFR* non-L858R subgroup within the *EGFR*-mutated group had a significantly higher OLI than the *EGFR* L858R subgroup did (**Figure 5B**). The *EGFR* non-L858R subgroup also had a higher OLI than the other-driver and negative groups (**Figure 5B**). All these differences also were observed in MOI comparisons; the *EGFR* 19del subgroup also had a significantly higher MOI compared with the *EGFR* L858R subgroup or the other-driver and negative groups (**Figure 5C**). However, no difference within the *EGFR*-mutated group was found in JI comparisons (**Supplementary Figure 10**). Given these results, we annotated the clonotypes of our patients using the VDJdb (36), McPAS-TCR (37), and TBAdb (38) databases. A median (range) of 7.45% (0%–44.83%) of clonotypes were annotated by VDJdb, and all were related to pathogens. Other databases had similar annotation rates: 7.04% (0%–40%) with McPAS-TCR and 5.50% (0.4%–65.50%) with TBAdb. Of these, only 9.52% and 7.87% of annotated clonotypes, in the respective databases, were related to cancer. The ratio of cancer-related TCRs to non–cancer-related TCRs was not associated with clinical characteristics and molecular characteristics, including age, smoking, tumor size, and *EGFR* mutations. Similarly, no difference was found in the *EGFR*-mutated group (**Supplementary Figure 12**).

To explore the prognostic value of the TCR similarity metrics, patients with stage I–III disease were observed. During a median follow-up of 416 days, five patients—two with stage I disease and three with stage III disease—experienced relapse. As shown in **Supplementary Figure 11**, three patients presented in the high OLI range, using the median OLI value (0.33) as the cut-off. The disease-free survival of patients with stage III disease was assessed in the high and low OLI groups according to the median OLI; no difference was found between the two groups (p=0.942; **Supplementary Figure 11**). Similarly, no difference was found when patients were divided into high or low JI (median, 0.023) or MOI (median, 0.185) groups on the basis of median values (p=0.771 and p=0.665, respectively; **Supplementary Figure 11**). Our data do not yet indicate whether TCR similarity metrics are associated with prognosis, and more population survival information is needed to confirm the prognostic value of the TCR repertoire.

Taken together, these findings indicate that the TCR similarity metrics were closely associated with clinical characteristics and gene mutations; low OLI or JI scores correlated with a favorable clinical status, suggesting that these indices may have the potential to predict patient prognosis.

## Discussion

The adaptive immune system is very important in fighting diseases, especially cancer. It is widely accepted that tumors are caused by the accumulation of genetic mutations and immune escape. Many studies have identified mutations in cancer genes, and some targeted drugs have been developed (39). Recently, the study of tumor immune escape and immune microenvironment has attracted considerable research attention. A better understanding of the immune microenvironment could help us better understand tumors and guide treatment (40). In this study, we performed TCR repertoire analysis in a cohort of 93 Chinese patients with lung cancer and reported the characteristics and clinical significance of TCR clonality, the Shannon index, and the OLI in lung cancer, especially in patients with early-stage disease.

The TCR repertoire has been investigated in several studies, and its relationship to immunotherapy in lung cancer has been shown (12, 13, 22). Recent studies have demonstrated changes in the TCR repertoire are associated with clinical parameters (13, 41). These studies showed that clonality was related to smoking and histology. Most of the smokers in our cohort (92.3%) had quit smoking, so we found no association between smoking and TCR. However, we did observe significantly higher clonality in patients who had quit smoking for less than 1 year or who still smoked compared with never smokers. The clonality was also higher in patients who had quit smoking for less than 1 year compared with those who had quit smoking for more than 1 year. Consistent with the results of Kargl et al., who found that the duration of smoking was positively correlated with clonality, our study confirms that duration of cessation may also affect clonality (41). The study by Kargl et al. also confirmed the correlation between TCR repertoire and histology; patients with squamous cell carcinoma had higher clonality and lower richness than patients with adenocarcinoma. However, no such difference was found in our study, even in disease stages I–III. Given that squamous cell carcinoma is associated with smoking (42, 43), the proportion of smokers in the patient population may have influenced the results. In our cohort, five of nine patients with squamous cell carcinoma were smokers, but four fifths of them had quit smoking for more than 6 months. Thus, the histology results may have been influenced by the proportion of smokers. Additional research is needed because of the limited number of squamous cell carcinoma patients in our cohort.

We demonstrated that clonality was positively correlated with pathological stage. Programmed death ligand 1, an effective biomarker for the prediction efficacy of immunotherapy in patients with advanced lung cancer, has no correlation with the efficacy of neoadjuvant or adjuvant immunotherapy in early-stage disease, indicating immunoheterogeneity between advanced and early-stage populations (44, 45). Similarly, clonality differences were observed between stage IV and stage I–III groups in our study. Studies have shown a negative correlation between tumor size and richness and no correlation between clonality and tumor size (13, 22). We also found no significant correlation between clonality and tumor size, although the larger tumor size group showed slightly higher clonality (p=0.06). The conflicting results of TCR diversity may be related to large number of patients with stage Ia disease in our cohort. Recent studies have suggested that CD8+ T-cell infiltration is weak in patients with early-stage lung cancer and that patients with positive CD8+ T-cell infiltration had a higher percentage of subclonal mutations (23, 46). When we excluded patients with stage Ia disease from analyses, a negative, though nonsignificant, correlation trend was found between the Shannon index and tumor size (r=−0.3343; p=0.150). However, this trend was not found in patients with stage Ia disease. Therefore, we hypothesized that larger tumor size is related to malignancy in stage I disease, so large size may cause more immune responses, such as inflammation, resulting in higher TCR diversity.

*EGFR*, as a common biomarker in lung cancer, was widely tested to guide clinical treatment. Recent studies have suggested that mutations in this gene may affect anti-tumor immune responses (34). The biology underlying the lower clinical response rates to immunotherapy in lung cancer with *EGFR* mutations has been investigated in several studies (13, 35) and is thought to be related to the higher diversity and lower clonality of patients with *EGFR* mutations. In our cohort, those with *EGFR* non-L858R mutations showed higher clonality, higher OLI scores, and lower Shannon index scores than other patients. No difference was found between *EGFR*-mutated and wild-type *EGFR* groups in our study, possibly because of the higher proportional distribution of patients to the *EGFR* non-L858R mutations group. The patients with *EGFR* L858R mutations also exhibited lower MOI scores compared with those in the *EGFR* 19del group. Previous studies have confirmed that patients with *EGFR* 19del benefit more from treatment with EGFR tyrosine kinase inhibitors versus patients with L858R mutations (47–49). Our results suggest that *EGFR* 19del tumors could better induce T-cell expansion or recruit T cells, which may induce the different response to EGFR tyrosine kinase inhibitors. However, recent studies have shown that patients with *EGFR* 19del have lower TMBs and poorer immunotherapeutic efficacy compared with patients who have *EGFR* L858R mutations and wild-type *EGFR* (50). In previous reports about *EGFR* status, most patients were not treated with first-line immunotherapy (50), and the TCR repertoire may have changed with treatment (14); thus, these previous reports do not conflict with our results. According to the higher TCR diversity associated with *EGFR* mutations (13) and our results, we propose possible explanations for higher clonality and MOI of *EGFR* 19del patients: Patients with *EGFR* 19del may have produced more bystander T cells because of inflammation and viral infection, for example; immune studies have shown that approximately 90% of infiltrating CD8+ T cells in patients with cancer are bystander T cells, unrelated to treatment (51, 52). Bystander T cells occupy the tumor space, where T cells are reportedly distributed spatially (28); thus, patients with *EGFR* 19del would have higher clonality, resulting in poorer immune treatment efficacy. However, no correlation was found between *EGFR* subtype and bystander TCR in annotation results in our study. Only approximately 7% of clonotypes were annotated in our study, so we believe that additional exploration of the composition of T cells by *EGFR* subtype will answer outstanding questions.

In addition, patient characteristics and the TMB may affect the efficacy of treatments (53–55). We found that OLI and JI scores were significantly associated with patient characteristics and TMB. By definition, a higher OLI or JI score means that greater numbers of tumor-specific infiltrating lymphocytes in tumor tissue are shared with postoperative peripheral blood. However, higher OLI and JI scores were detected in older patients, those with larger tumors, smokers, and those with higher TMBs—all of which were associated with poorer prognosis—but these relationships (except smoking) were not established with the MOI. Given the definition of the three metrics, these results might suggest that more low-frequency clonotypes were found in the blood of these patients, whereas previous studies have suggested that these low-frequency TCRs were bystander T cells (20, 56). The correlation of OLI score and the CD8+CD28+ ratio in CD8+ T cells may support our view that higher OLI may be related with more bystander T cells. In non–small-cell lung cancer, higher peripheral proliferating CD8+ T cells and lower CD4+ T cells, compared with healthy controls, have been observed (33, 57). As an essential co-stimulatory, CD28 on CD8+ T cells interacts with B7 molecules on antigen-presenting cells to activate the anti-tumor immune response of CD8+ T cells to tumor antigens (58). Several previous investigations showed that the high levels of peripheral CD8+CD28+ T cells were linked to better prognosis (33, 58–60). In non–small-cell lung cancer, Liu et al. revealed that high levels of CD8+CD28+ T cells in peripheral blood before anti-tumor treatment (chemotherapy/radiation/immunotherapy/surgery) were associated with prolonged progression-free survival and overall survival (58, 60). This negative correlation between OLI and CD8+CD28+ suggests that patients with high OLI scores may have poorer survival. According to our follow-up results, 60% of patients who experienced relapse were assigned to the high OLI group, indicating that TCR similarity is a potential prognostic factor. Additional follow-up studies are needed to confirm these findings.

Our study has several limitations. First, our results are limited by the retrospective nature of our analysis; the relationship between TCR similarity metrics and prognosis must be verified in prospective studies. Second, the hypothesis of bystander T cells with regard to *EGFR* 19del was not supported by our annotated results and MOI assessment; additional exploration of the composition of T cells in *EGFR* subtypes will improve understanding about the mechanism of TCR repertoire differences among *EGFR* subtypes. Moreover, dynamic changes in the TCR repertoire are lacking.

## Conclusions

In conclusion, this study offers novel evidence that TCR repertoires are related to tumor status and gene mutation and that different *EGFR* mutation subtypes are correlated with TCR clonality and the Shannon index. Furthermore, the TCR similarity metrics correlated with several clinical characteristics and with tumor mutation status. These results could improve understanding of the immune microenvironment of tumors and have the potential to guide treatment and prognostic assessment of patients.

## Data Availability Statement

The original contributions presented in the study are included in the article/**Supplementary Material**, and the original data presented in the study are deposited in the Figshare (https://figshare.com/) repository, accession number (https://doi.org/10.6084/m9.figshare.14112080.v1). Further inquiries can be directed to the corresponding authors.

## Ethics Statement

This study was approved by the Ethic Committee of Peking Union Medical College Hospital. All patients provided written informed consent to participate in this study.

## Author Contributions

NL, ZC, SL, and ZJ were involved in the conception and design of the study. HY and ZJ were major contributors in developing the manuscript. HY, ZJ, XY, PW, YDW, and YYW collected and analyzed the clinical information. DG, RC, and XX were in charge of the statistical analysis of data of T-cell receptor repertoire. All authors contributed to the article and approved the submitted version.

## Funding

This study was supported by the Chinese Academy of Medical Sciences Young Medical Talent Award Fund (No. 2018RC320005), the Beijing Natural Science Foundation (No. 7182132), the Beijing Students’ Platform for Innovation and Entrepreneurship Training Program (No. 2019zlgc0629), and the National Key Research and Development Program of China (No. 2016YFC0901500).

## Conflict of Interest

DG, RC, and XX are employees of Geneplus-Beijing Ltd.

The remaining authors declare that the research was conducted in the absence of any commercial or financial relationships that could be construed as a potential conflict of interest.

## Abbreviations

TCR, T-cell receptor; TMB, tumor mutational burden; CDR3, complementarity-determining region 3; OLI, overlap index; JI, Jaccard index; MOI, Morisita index.
