# Supplementary material for: Characteristics of T-Cell Receptor Repertoire and Correlation With EGFR Mutations in All Stages of Lung Cancer
Source: Front Oncol. 2021 Mar 11;11:537735. doi: 10.3389/fonc.2021.537735 (PMC7991722; doi:10.3389/fonc.2021.537735)
Supplement: Supplementary file 1 [file DataSheet_1.docx]

Supplementary Material

**Supplementary Figures 1–12**

**
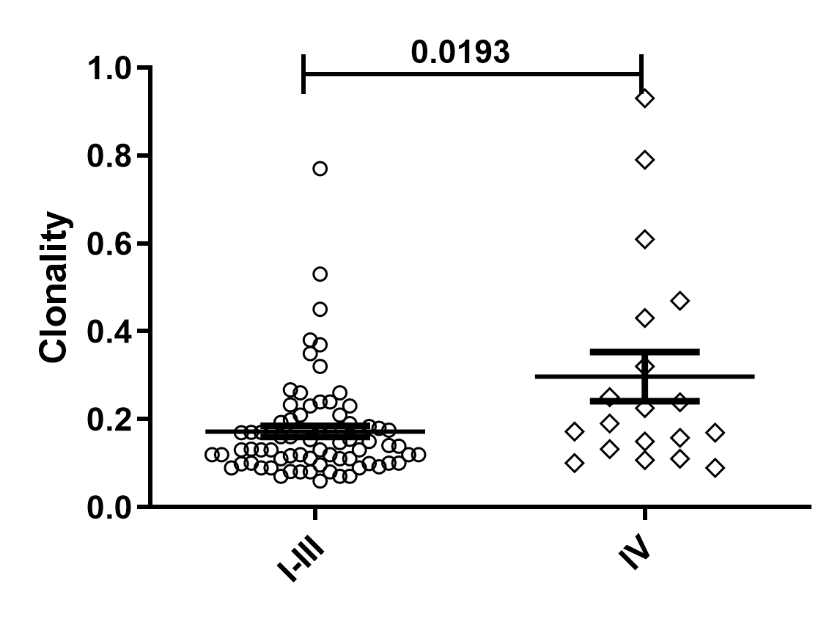
**

**Supplementary Figure 1.** Differences in clonality between patients with non-distant metastasis and those with distal metastasis. Statistical analysis was performed using the Mann-Whitney test. Bar lines indicate medians, and whiskers represent the 5th–95th percentiles. *p<0.05.

**
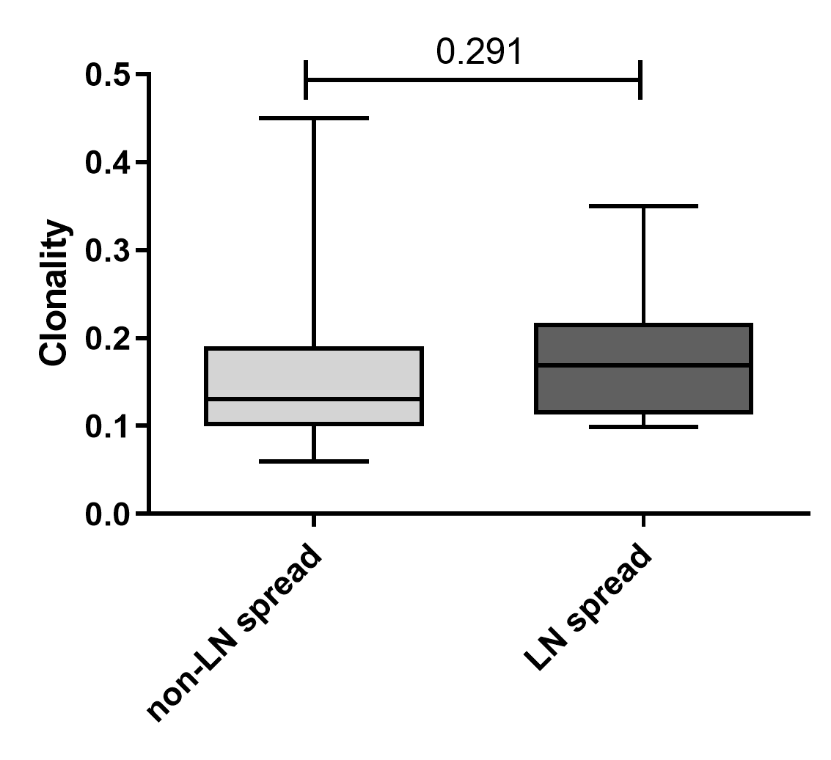
**

**Supplementary Figure 2.** Differences in clonality between patients without lymph node (LN) metastasis (non-LN spread) and those with LN metastasis (LN spread) in the population without distal metastasis. Statistical analysis was performed using the Mann-Whitney test. Bar lines indicate medians, and whiskers represent the 5th–95th percentiles.

**
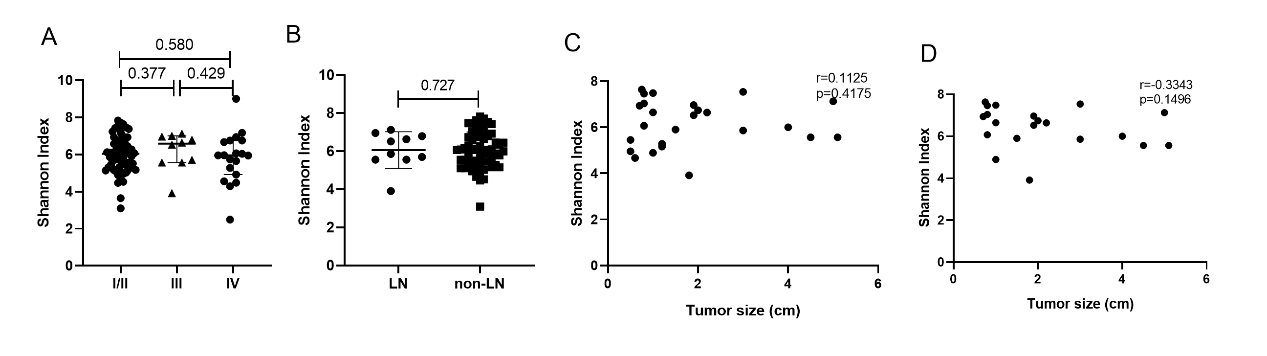
**

**Supplementary Figure 3. (A)** Comparison of Shannon index between different pathological stages. (**B**) Comparison of Shannon index between patients with versus without lymph node metastasis. (**C**) Correlation between tumor size and Shannon index of patients without lymph node metastasis. **(D)** Correlation between tumor size and Shannon index in patients with stage Ia disease. Statistical analysis was performed using the Mann-Whitney test and Spearman’s rank test. Bar lines indicate medians, and whiskers represent the 5th–95th percentiles. *p<0.05.


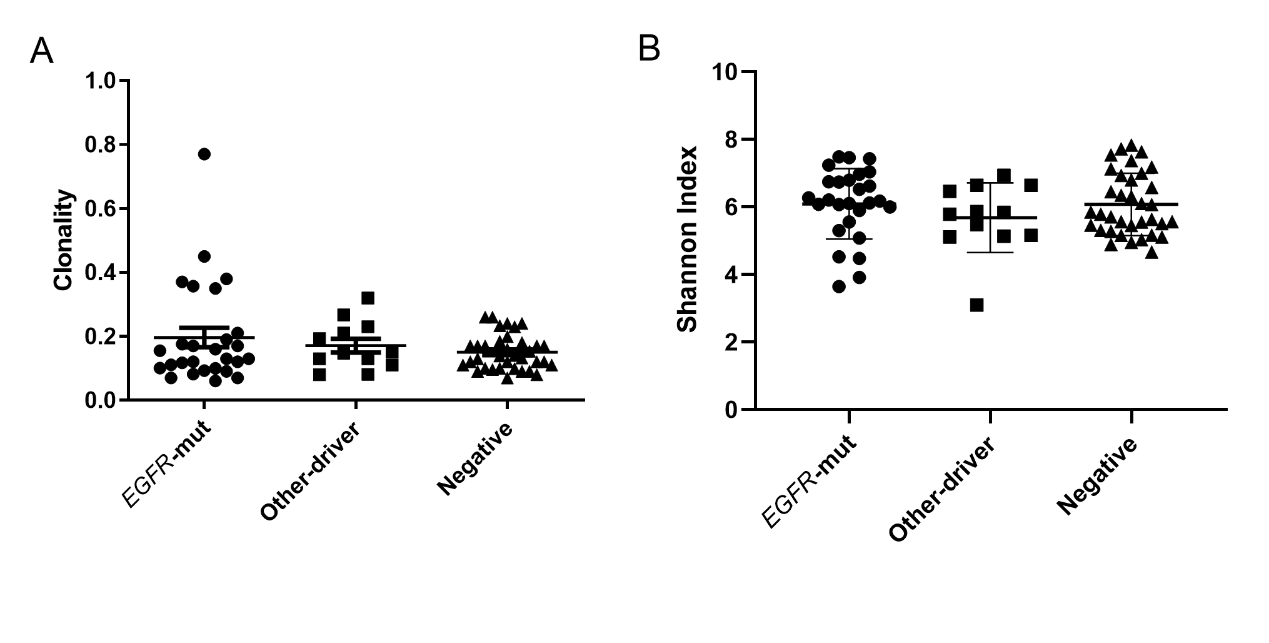


**Supplementary Figure 4. (A)** Differences of clonality in the three groups of patients with stages I–III disease. **(B)** Differences of Shannon index in the three groups of patients with stages I–III disease. Statistical analysis was performed using the Mann-Whitney test. Bar lines indicate medians, and whiskers represent the 5th–95th percentiles.

**
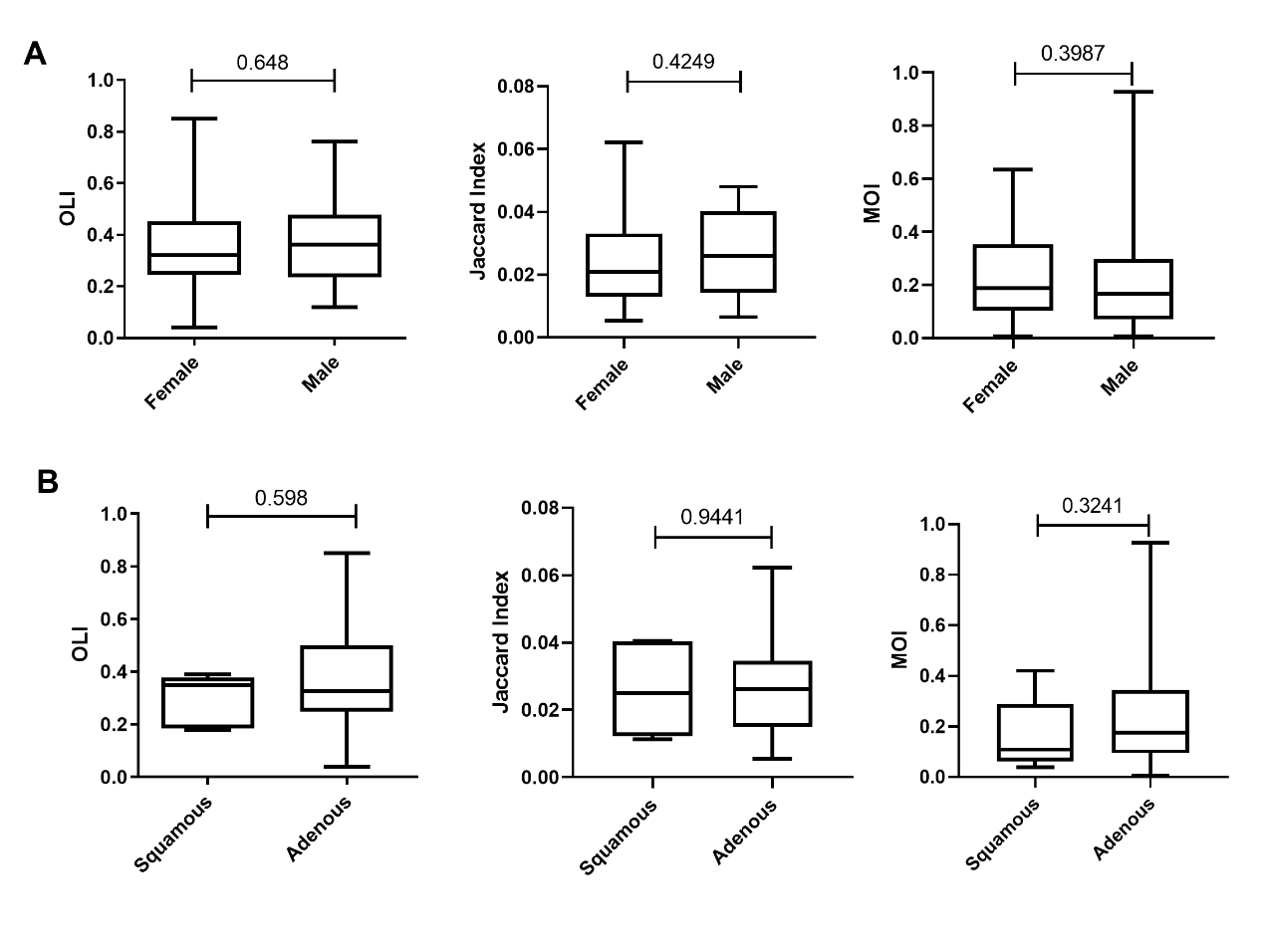
**

**Supplementary Figure 5.** T-cell receptor (TCR) similarity metrics compared according to sex and histologic subtype. **(A)** Comparison of TCR similarity metrics between male and female patients. **(B)** Comparison of TCR similarity metrics between patients with adenocarcinoma (adenous) and squamous carcinoma. Statistical analyses were performed using the Mann-Whitney test and Spearman’s rank test. Bar lines indicate medians, and whiskers represent the 5th––95th percentiles. MOI, Morisita Index; OLI, Overlap Index.

**
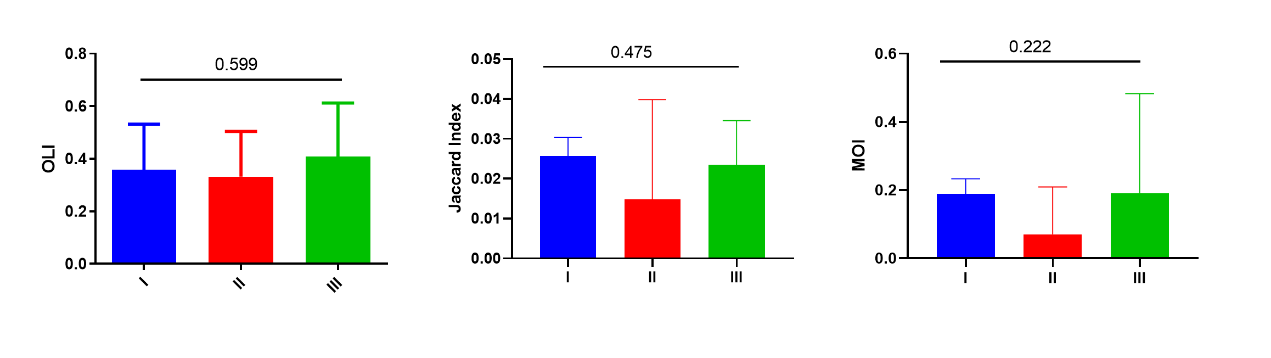
**

**Supplementary Figure 6.** T-cell receptor similarity metrics compared according to pathological stage. Statistical analysis was performed using the one-way ANOVA test. Bar lines indicate medians, and whiskers represent the 5th–95th percentiles. MOI, Morisita Index; OLI, Overlap Index.

**
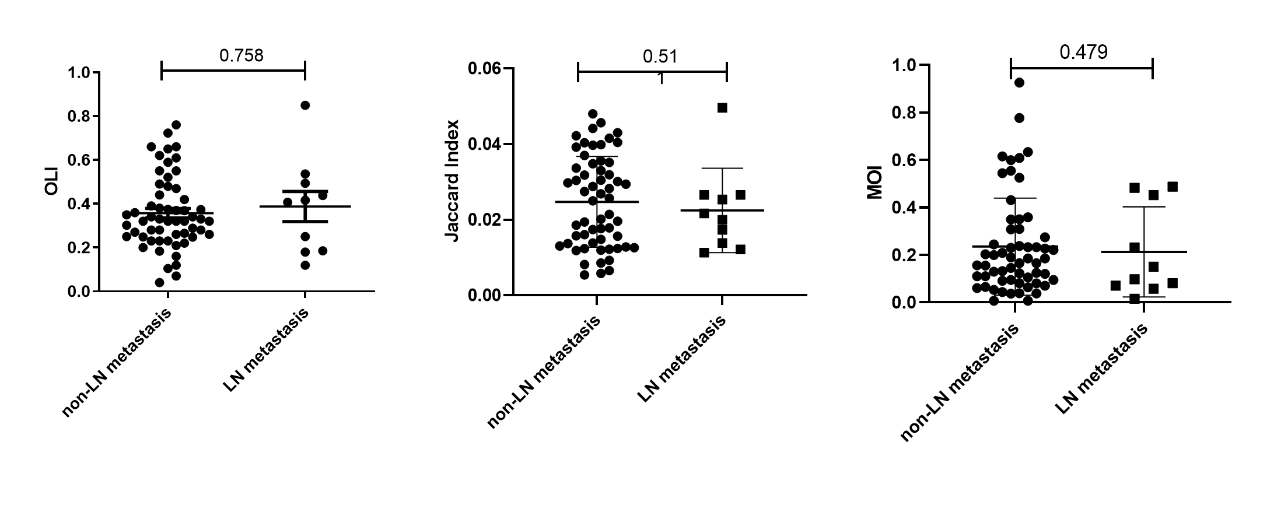
**

**Supplementary Figure 7.** Differences in T-cell receptor similarity metrics between patients without lymph node (LN) metastasis and those with LN metastasis. Statistical analysis was performed using the Mann-Whitney test. Bar lines indicate medians, and whiskers represent the 5th–95th percentiles. MOI, Morisita Index; OLI, Overlap Index.

**
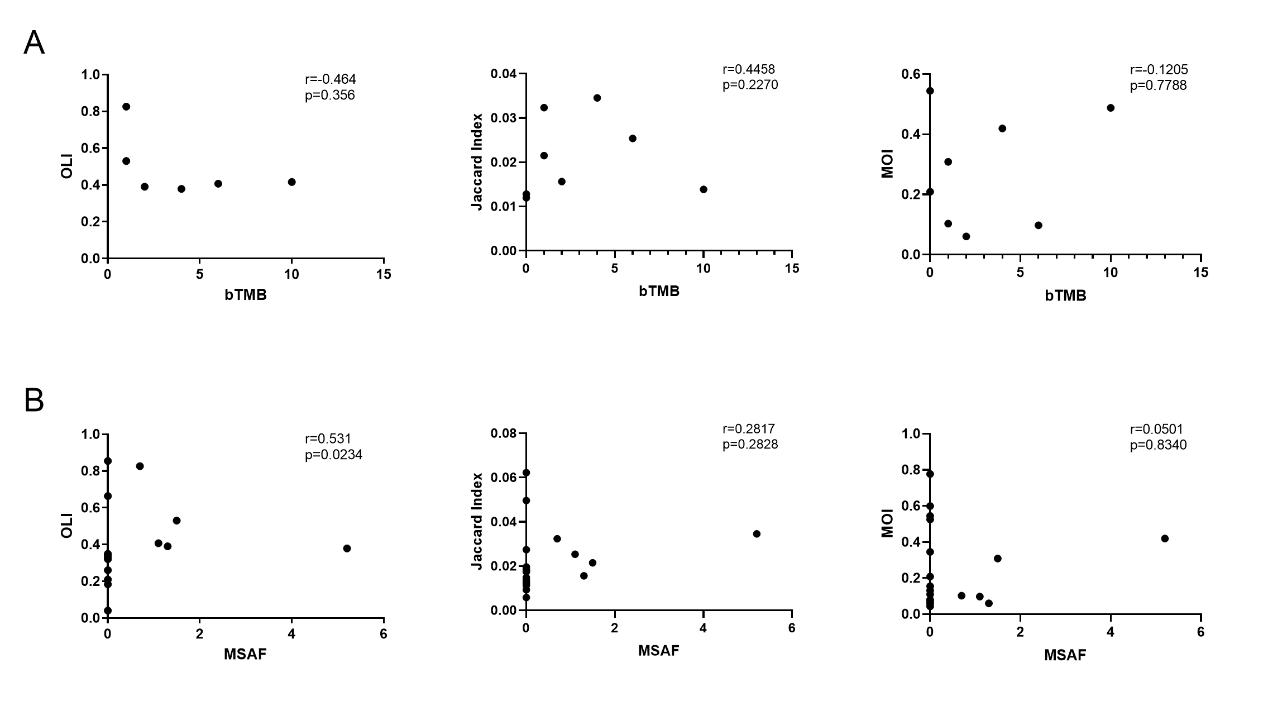
**

**Supplementary Figure 8. (A)** Correlation between T-cell receptor (TCR) similarity metrics and tumor mutation load of blood (bTMB). **(B)** Correlation between TCR similarity metrics and maximum somatic allele frequency (MSAF). Statistical analysis was performed using Spearman’s rank test.

**
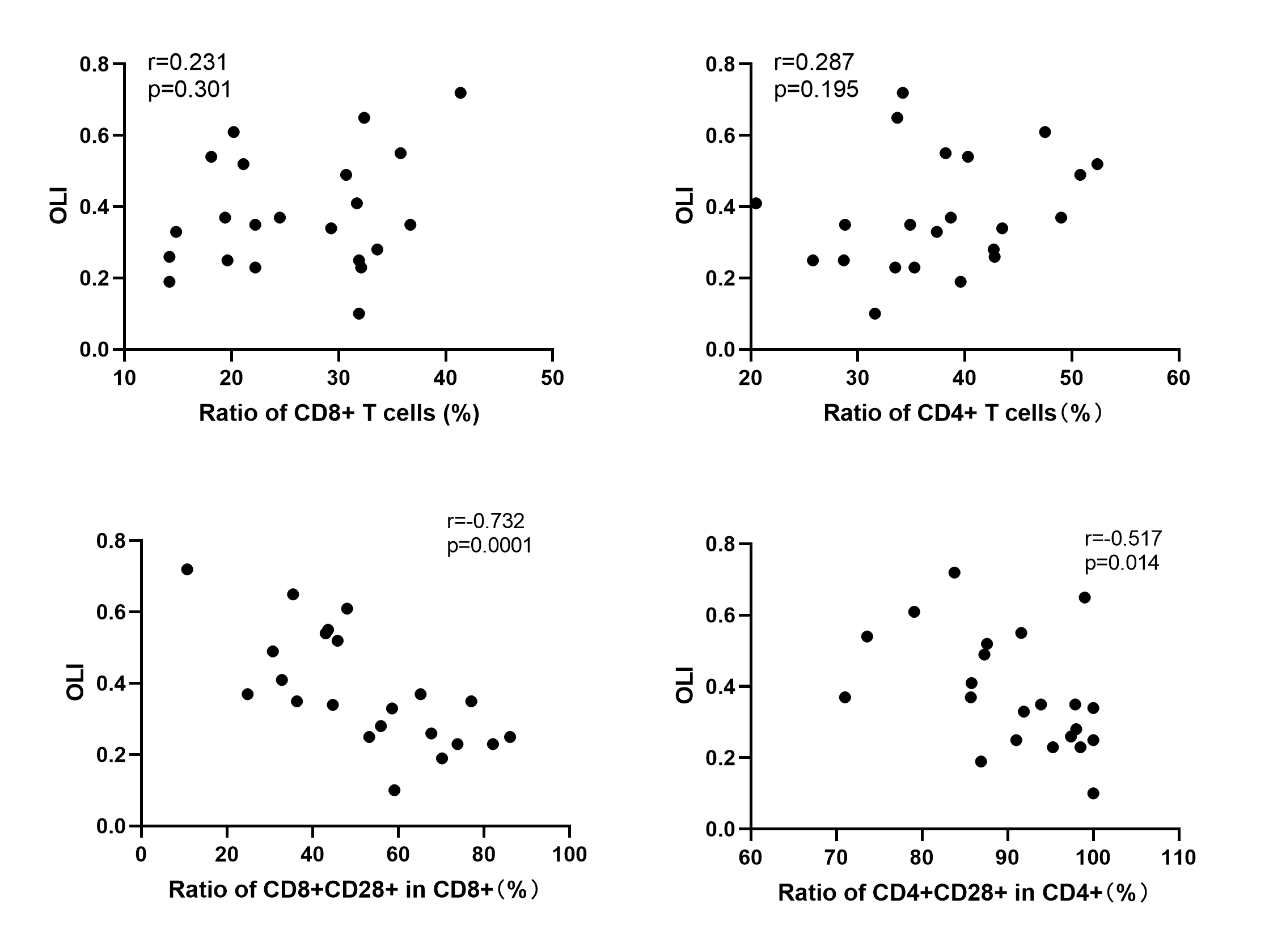
**

**Supplementary Figure 9.** Correlation between the Overlap Index (OLI) and flow cytometry. Statistical analysis was performed using Spearman’s rank test.

**
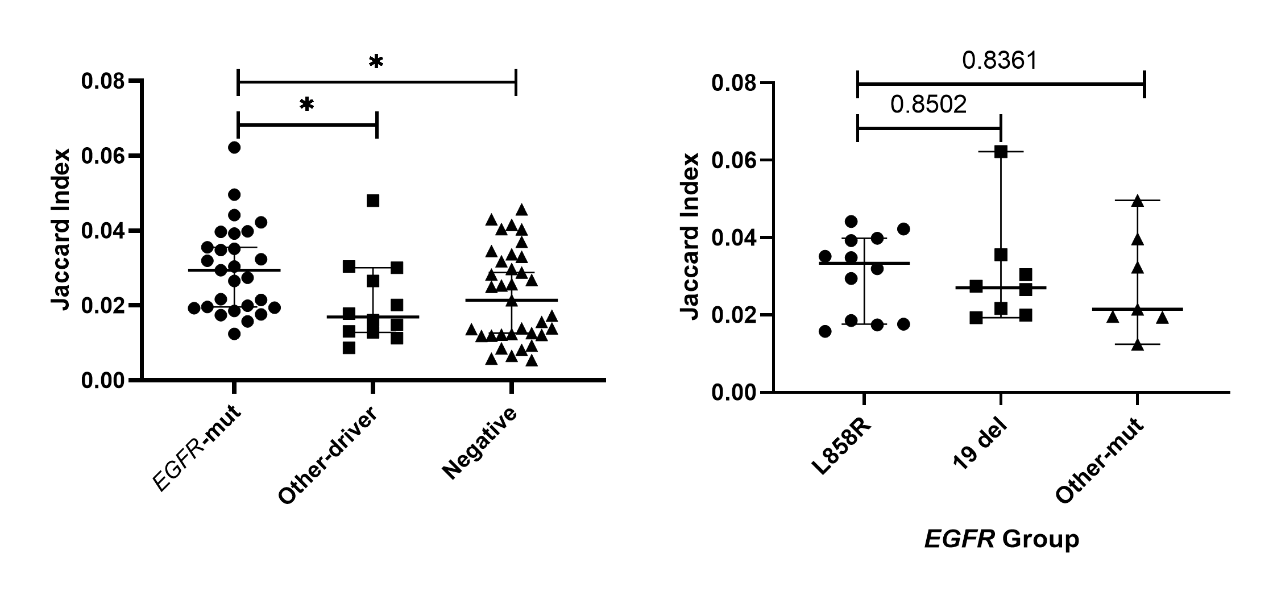
**

**Supplementary Figure 10.** Difference of Jaccard index **(A)** among *EGFR*, other-driver, and negative groups and **(B)** among *EGFR* subtypes. Statistical analysis was performed using Spearman’s rank test and the Mann-Whitney test. Bar lines indicate medians, and whiskers represent the 5th–95th percentiles., mutation.

**
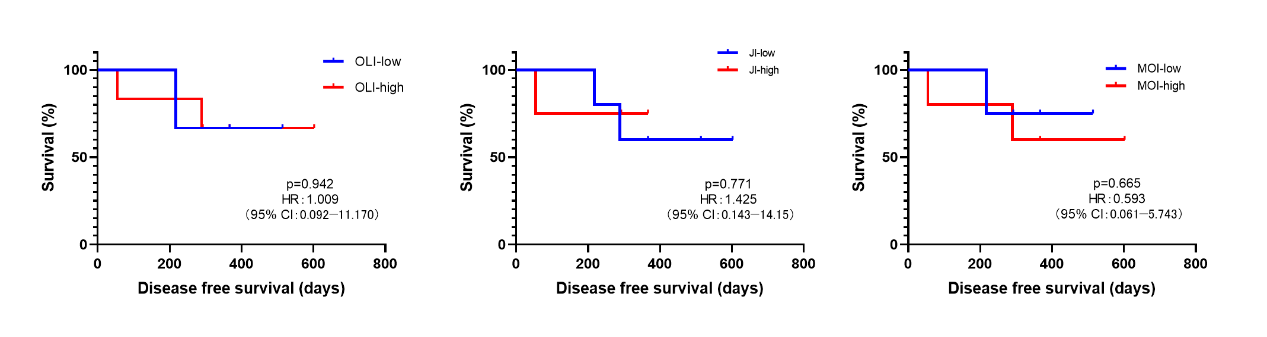
**

**Supplementary Figure 11.** T-cell receptor repertoire distribution of patients and survival of patients with stage III disease in this study. Disease-free survival of patients with stage III disease in different Overlap Index (OLI) levels (OLI cut-off, 0.33). Survival analysis was performed using the log-rank (Mantel-Cox) test. HR, hazard ratio; JI, Jaccard Index; MOI, Morisita Index.


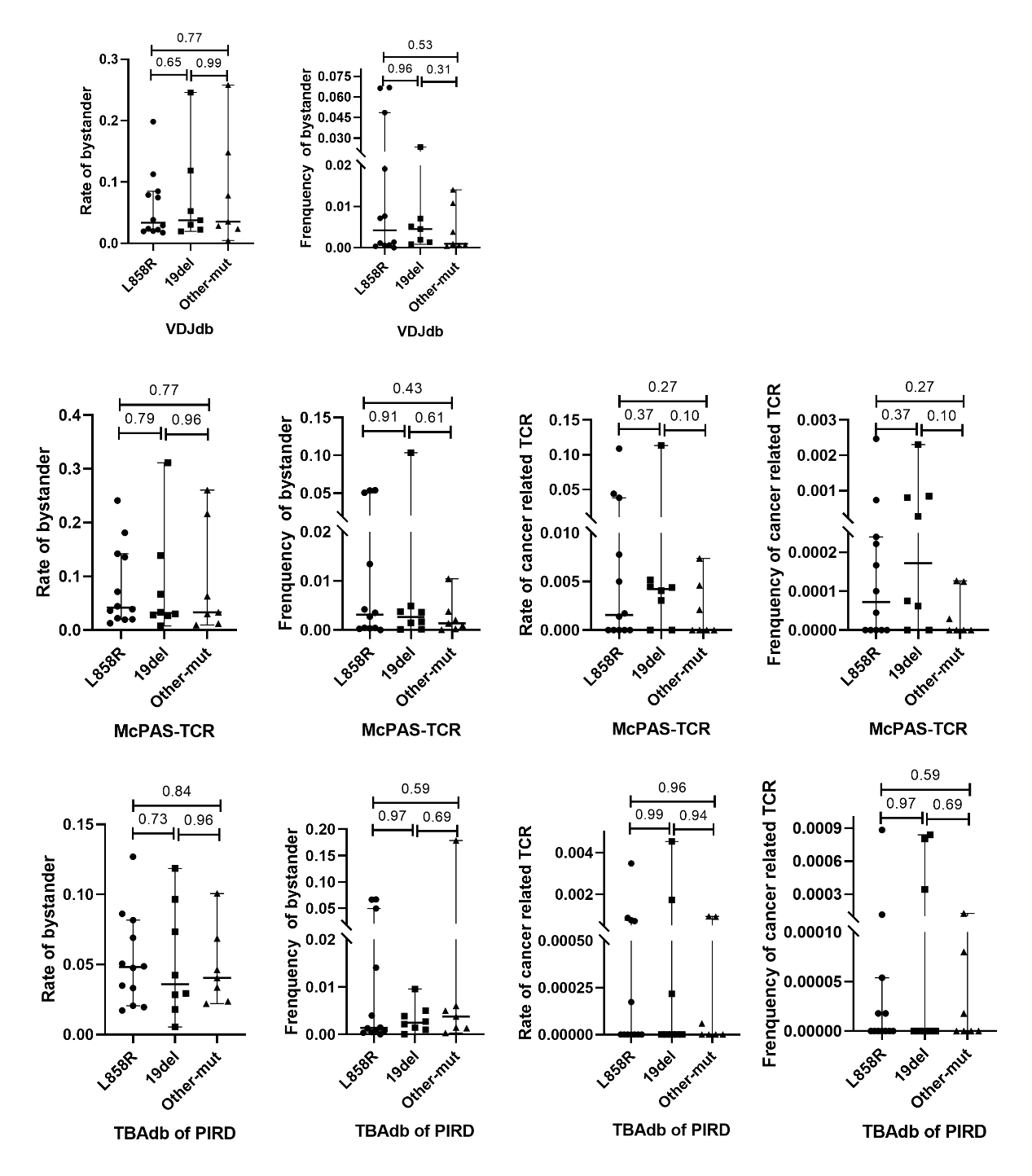


**Supplementary Figure 12.** The correlation of *EGFR* subtypes and “bystander” or cancer-related T-cell receptors (TCRs)in VDJdb (top), McPAS-TCR (middle), and TBAdb (bottom) databases. Rate indicates the ratio of annotated clonotypes with all clonotypes of patients. Frequency indicates the sum of frequencies of annotated clonotypes. Statistical analysis was performed using Spearman’s rank test. Bar lines indicate medians, and whiskers represent the 5th–95th percentiles. Mut, mutation.
